# Mr. Hammick's Case of Gun-Shot Wound

**Published:** 1812-04

**Authors:** A. Baird

**Affiliations:** Earl St. Vincent's, Rockelts, Essex


THE
### Medical and Physical Journal.

4 OF VOL. XXVII.] APRIL, 1812. [NO. 158.

*To the Editors of the Medical and Physical Journal.*

GENTLEMEN,

I HAVE received the inclosed interesting case from Mr. Hammick, senior surgeon of the Plymouth Royal Hospital, with his particular desire that it may be inserted in your valuable Journal. By giving it a place you will also oblige

Your's, &c.

*Earl St. Vincent's,* A. BAIRD, M.D.

*Rocketts, Essex. Feb.* 28, 1812.

Mr. W. E. of the Royal Navy, aged about 33, of a full plethoric habit, and of a vigorous healthy constitution, first lieutenant of his Majesty's frigate Leonidas, then lying in Hamoaze, was admitted into the Royal Naval Hospital at Plymouth, under my care, about nine o'clock in the morning of the 9th of October, 1811. On my first visit, I received from him, and the surgeon who accompanied him on shore, the following particulars: That he had, about two hours before, in a temporary fit of insanity, (having been quite well late the preceding night,) put far back into his mouth a loaded pistol, and in that situation had fired it off; the shock threw him off his legs, and he fell forwards on his face in his own cabin, where his messmates immediately found him, and prevented his further injuring himself by the removal from his table of a loaded pistol and an unsheathed razor; in a few minutes he was so far recovered from the explosion, as to be able to relate what he had done; in a short time afterwards he was put into a boat and conveyed to the hospital. I further learnt, that, as the frigate was repairing in Hamoaze, all the ammunition had been sent on shore, according to the regulations of the service, excepting a small quantity for the use of the marines; that he had early that morning directed the sergeant of marines to make him sixteen cartridges for ship's pistols, with one of which he had fired the pistol, but, not having a ball large enough for a ship's pistol, he had put in one of a smaller size. Previous to his admission here he had bled very freely from the mouth, and had been constantly coughing, and spitting up clotted blood, not having the power of swallowing it.

On examination, I found that his face and lips were much swollen and greatly discolored, particularly on the right side; that there was a pretty free discharge of blood issuing from the mouth, mixed with saliva; that, although he was able to close the jaws, it was done with some difficulty, and increased the sense of suffocation; that the jaws and teeth were perfect, proving the correctness of his statement of having put the pistol fairly into his mouth before he had fired it; the tongue, inner side of the cheeks, roof of the mouth, and soft palate, were burnt as black as if they had been pitched over. The uvula was much lacerated, and hung down; and a little to the right side, and above it, I could pass my finger through a small opening in the right palate-bone, which was no doubt made by the passage of the ball; but, as to what had become of it, and the cartridge-paper, I was completely in the dark. He could move his neck with freedom, which was beginning to swell, and becoming emphysematous; and he had freely the rotatory motion, as well as the flexion and extension of the head. His cough was very distressing, from the lacerated state of the throat and pharynx, and from the coagulated blood getting down without his having the power of swallowing; his voice was weak and low, and it was a fatigue to speak; the pulse was feeble, and he felt cold, caused not only from exposure in the boat on coming on shore, but also from the sense of chilliness and horror which almost invariably seizes persons immediately on the infliction of any wound by fire-arms, even in those of the most undaunted courage; he was perfectly sensible, thoroughly convinced of the impropriety of the act he had committed, was very anxious to live, but from his feelings was confident he should not survive the wound.

Some infusion of senna was now ordered, but we were soon convinced, from the convulsive agony the effect to swallow it produced, that all attempts of that sort must for the present be abandoned; indeed, his sufferings at this trial were such as gave us the idea that some part of the fluid had got into the trachea; and he was heard afterwards to declare, that he suffered more at this attempt than from any thing that ever was done at any period of his cure. A cooling wash was ordered to the outside of his neck and face, and he was desired to gargle the mouth and throat very frequently, which he was able to do, although he had not the power of forcing a single drop down the œsophagus.

In the evening, I found that he had passed a miserable day, could not permit himself for a moment to recline in bed, was incessantly coughing, his face and neck were much swelled and discolored, the latter being very emphysematous, the lacerated state of the pharynx permitting the free escape of air into the cellular membrane and interstices of the muscles of the neck. Fever now coming on, with fullness of the pulse, twenty ounces of blood were taken from the arm, a purgative clyster was thrown up, and he continued to gargle, either with lemonade, which was grateful, or the common detergent gargle.

10th. After having gone through a night of great suffering, as to suffocation, burning pain of the inward parts, and incessant cough, we found him with all his symptoms increased, excepting that the pulse was not quite so full as the night before. He persisted in the use of the gargle, and had another clyster, which answered very well. About three o'clock in the afternoon, during a violent fit of coughing, in which he thought he should have been suffocated, he threw up the ball, with some slough and cartridge-paper; from which, however, he did not experience so much relief as we might have expected. The ball, which I now have, is a small pistol-ball, having on one side a flat mark as if made by its passage through the palate-bone, after which I suppose it fell, together with the paper, and was prevented passing down the œsophagus by the lacerated state of the pharynx; for, had the ball found its way into the stomach, it would have been discharged by the anus, instead of being thrown up by the mouth. At the evening visit, all that we could say he had gained by the discharge of the ball, was some relief from the fullness which he had complained of about the middle of the neck, none of the other symptoms were alleviated: on the contrary, he said his head was splitting, and his thirst insupportable, with a pungent heat of the skin. I therefore directed the nurse to throw up a pint of cold and weak lemonade as a clyster, which, in about half an hour, relieved his thirst, and cooled his body so much that he begged to have it repeated; and it was ordered he should have it as often as he liked during the night.

11th. This morning he was much cooler; head-ach less, and thirst very considerably reduced, in consequence of the lemonade injections, of which, according to his own desire, the nurse had given him seven in the last twelve hours; but he had struggled through a night of harrassing and distressing cough, discharging sloughs and clots of blood. His face and neck were now so increased, particularly the latter, and so much discolored, as gave him a most ghastly appearance; the air crackling under the finger in every direction. He complained much of uneasiness in the region of the stomach. In the evening, after some efforts, I succeeded in getting a tube well down the œsophagus, and threw into the stomach about a pint of tea, which was particularly pleasant, and soon took off the pain and sinking, as he described it, of the epigastric region; but he still requested to have the cold lemonade clysters, as he said we could not form any

 conception conception how grateful they felt, and how they always relieved his thirst and cooled his body.

12th. Had passed a night of great suffering, for the lacerated and sloughing state of the throat and mouth left him not a moment free from cough, and the fits of coughing were the most distressing I ever witnessed, and more than once thought he would have expired. This morning the appearance of his neck and throat were really frightful from the extent of the swelling and discoloration. By means of the tube I got at one time some milk, and a few hours afterwards some lemonade, into the stomach, and we went on with our gargles and cold clysters.

13th. Having had a rather better night, we had hopes that he might recover, and from this morning he began to amend somewhat; but he never swallowed a drop of any thing till the morning of the 19th, when he was delighted to tell me that he thought a few drops of tea had gone down. From this period, viz. the 19th, his power of swallowing increased so rapidly, that by the 22d we were enabled to lay aside giving either nourishment or drinks by the tube, for up to this period I had constantly passed it twice or three times a day, as he felt inclined to receive nourishment. After the 24th the amendment was so progressive and flattering, that of course it would only lead to a tiresome and long detail to give from this moment the daily occurrences. The sloughs daily threw off, and the cough lessened. The swelling and discoloration of the neck gradually subsided, and by the 10th of November he was comfortable as to his feelings, only he had not the power of freely moving the lower jaw, nor of opening it to any extent. The right side of the face still kept up a good deal of its swelling, and at one time threatened to break, but on the 23d of November a profuse ptyalism came on, which continued more or less nearly a fortnight, and from that moment the face by degrees lost its hardness, pain, and swelling. We may conclude, therefore, that this affection of the face had been kept up by the extremity of the parotid duct into the mouth, having been closed by the accident; but, the sloughs having now thrown off, the passage was clear so as to allow the saliva freely to pass. After this his progress was most rapid, so that by the 13th of December he was discharged the hospital quite cured and well. The only difficulty he experienced was a degree of mucus spitting, or cough. The uvula was contracted and drawn towards the right side, and he was obliged to be cautious not to attempt swallowing any food that was not very tender and well masticated. The right articulation of the lower jaw was a little stiff, his voice somewhat somewhat weaker than before, and pain was felt in the teeth when exposed to the cold air, or in taking any thing cold into the mouth. Excepting in these particulars, which in a few weeks will be most probably overcome, he felt perfectly well, and free from all inconvenience from the wound.

STEPHEN LOVE HAMMICK.

*Royal Hospital, Plymouth,*
*January,* 1812.

P.S. Finding that it would be impossible to pass the tube commonly used in getting fluids into the stomach, I took a moderate sized elastic gum catheter, one that perfectly fitted the elastic gum bag of a common hydrocele apparatus, then curving the catheter so as to make the turn over the tongue into the œsophagus with the flexible stilette in it. I generally succeeded very easily in introducing it, but I found that, when the catheter had passed a certain distance, it was better to withdraw the stilette by degrees, gradually passing on the catheter, inclining it towards the left side till it was fairly down its whole length, so that the upper end was only just without the teeth when the stilette was totally withdrawn; and then, by fixing the nosle of the hydrocele bag, I was able to throw with the greatest ease the fluid into the stomach; and I soon discovered that by leaving the tongue alone to nature the tube passed better; for at times, when we used a spoon either to depress the tongue or attempt to draw it forwards, the epiglottis being raised by the exertion of the patient, our tube more than once slipped into the trachea, producing distressing sensations, all which inconveniences were avoided by allowing the tongue to be free.

